# Kir6.2 activation by sulfonylurea receptors: a different mechanism of action for SUR1 and SUR2A subunits via the same residues

**DOI:** 10.14814/phy2.12533

**Published:** 2015-09-28

**Authors:** Maria A Principalli, Julien P Dupuis, Christophe J Moreau, Michel Vivaudou, Jean Revilloud

**Affiliations:** 1 Institut de Biologie Structurale (IBS), University of Grenoble AlpesGrenoble, France; 2 CNRS, IBSGrenoble, France; 3 CEA, IBSGrenoble, France; 4 Institut Interdisciplinaire de Neurosciences CNRS UMR 5297Bordeaux, France

**Keywords:** ATP-sensitive K+ channels, Diazoxide, functional coupling

## Abstract

ATP-sensitive potassium channels (K-ATP channels) play a key role in adjusting the membrane potential to the metabolic state of cells. They result from the unique combination of two proteins: the sulfonylurea receptor (SUR), an ATP-binding cassette (ABC) protein, and the inward rectifier K^+^ channel Kir6.2. Both subunits associate to form a heterooctamer (4 SUR/4 Kir6.2). SUR modulates channel gating in response to the binding of nucleotides or drugs and Kir6.2 conducts potassium ions. The activity of K-ATP channels varies with their localization. In pancreatic *β*-cells, SUR1/Kir6.2 channels are partly active at rest while in cardiomyocytes SUR2A/Kir6.2 channels are mostly closed. This divergence of function could be related to differences in the interaction of SUR1 and SUR2A with Kir6.2. Three residues (E1305, I1310, L1313) located in the linker region between transmembrane domain 2 and nucleotide-binding domain 2 of SUR2A were previously found to be involved in the activation pathway linking binding of openers onto SUR2A and channel opening. To determine the role of the equivalent residues in the SUR1 isoform, we designed chimeras between SUR1 and the ABC transporter multidrug resistance-associated protein 1 (MRP1), and used patch clamp recordings on *Xenopus* oocytes to assess the functionality of SUR1/MRP1 chimeric K-ATP channels. Our results reveal that the same residues in SUR1 and SUR2A are involved in the functional association with Kir6.2, but they display unexpected side-chain specificities which could account for the contrasted properties of pancreatic and cardiac K-ATP channels.

## Introduction

ATP-sensitive potassium channels (K-ATP channels) allow potassium ions to cross-selectively cell membranes as a function of the internal ATP/ADP ratio (Noma 1983; Ashcroft et al. 1988; Nichols et al. 1996). While ATP inhibits the channels, MgADP acts as a physiological opener. Because of this unique regulation by intracellular nucleotides, K-ATP channels are usually considered as direct sensors of the metabolic state of the cell. In pancreatic *β*-cells, where their physiological function is best understood, they couple the cytoplasmic concentration of nucleotides levels to the insulin secretion machinery via the modulation of the membrane voltage. K-ATP channels are expressed in other organs and tissues, including the brain, the heart, the skeletal, and smooth muscles where they contribute to protection against acute metabolic stress (Seino and Miki 2003; Kane et al. 2005). Pharmacologically, K-ATP channels are the target of inhibitors such as sulfonylureas (Gribble and Ashcroft 1999), and potassium channels openers (KCO) such as Diazoxide (Moreau et al. 2000, 2005a,b), which are commercialized as type 2 diabetes and antihypertensive vasodilator medications, respectively.

**Figure 1:**
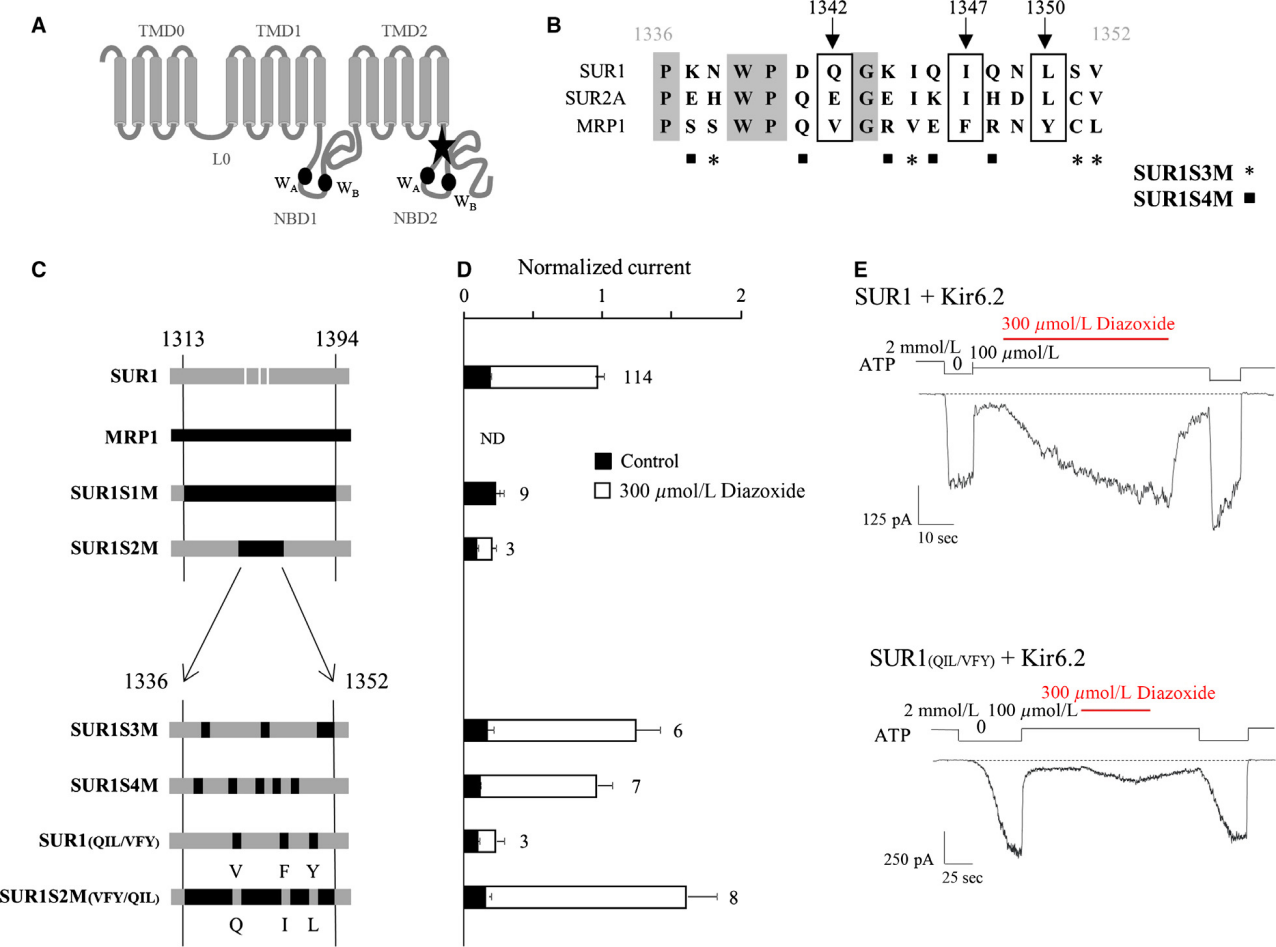
SUR1 residues Q1342, I1347, and L1350 are essential for K-ATP channel activation by Diazoxide. (A) putative membrane topology of SUR1 (NBD, nucleotide-binding domain; WA, Walker A motif; WB, Walker B motif). The star indicates the region identified by Rainbow et al. (2004) and mutated to obtain the S1M chimera. (B) alignment of the amino acid sequences of human SUR1, rat SUR2A, and human MRP1. Three residues (boxed) are homologous in SURs and not in MRP1: Q1342, I1347, and L1350. Stars and squares indicate the MRP1 residues introduced in SUR1 to obtain SUR1S3M and SUR1S4M, respectively. (C) schematic representation of the chimeras. SUR1 and MRP1 elements are drawn in gray and black, respectively. For clarity, residues Q1342, I1347, and L1350 of SUR1 are indicated by white stripes. The amino acid composition of the constructs were as follows: SUR1S1M = SUR1(M1-V1313) + MRP1(V1261-F1341) + SUR1(R1394-K1582); SUR1S2M = SUR1(M1-P1336) + MRP1(P1284-L1300) + SUR1(V1352-K1582); SUR1S3M = SUR1 with mutations N1338S, I1345V, S1351C, and V1352L; SUR1S4M = SUR1 with mutations K1337S, D1341Q, K1344R, Q1346E, and Q1348R; SUR1(QIL/VFY) = SUR1 with mutations Q1342V, I1347F, and L1350Y, SUR1S2M(VFY/QIL) = SUR1S2M with mutations V1290Q, F1295I, and Y1298L. (D) Diazoxide responses were measured in inside-out patches excised from oocytes coexpressing Kir6.2 and wild type or chimeric SURs or MRP1 as indicated. Diazoxide (300 *μ*mol/L) was applied in the presence of 100 *μ*mol/L ATP, and currents were normalized to the current measured in the absence of nucleotides immediately before opener application. Application of 100 *μ*mol/L ATP alone (black bars) was used as a control. Numbers at right of bars indicate the number of patches included in each average. (E) Representative patch-clamp recordings illustrating the responses of wild type and SUR1_(QIL/VFY)_ channels to 300 *μ*mol/L Diazoxide in the presence of 100 *μ*mol/L ATP. SUR1, sulfonylurea receptor 1; MRP, multidrug-resistance associated protein.

From a structural point of view, K-ATP channels result from the association of two different proteins: the sulfonylurea receptor (SUR), which belongs to the ATP-binding cassette (ABC) protein family, and the inward rectifier potassium channel Kir6 (Aguilar-Bryan et al. 1995; Inagaki et al. 1995a). Four pore-forming Kir6 subunits are arranged at the center of the complex and surrounded by four regulatory SUR subunits, generating a macromolecular assembly of about 950 kDa (Clement et al. 1997; Mikhailov et al. 2005). The presence of endoplasmic reticulum retention signal on both SUR and Kir6 ensures that only properly assembled channels reach the plasma membrane (Zerangue et al. 1999). SUR presents strong homologies with other eukaryotic ABC transporters, even though no transport activity has been reported for this protein. SUR retains the typical architecture of ABC transporters, with two transmembrane domains (TMD1 and TMD2) and two cytoplasmic nucleotide-binding domains (NBD1 and NBD2; Fig. 1A), plus an additional N-terminal TMD0. Importantly, TMD0 has been shown to play a crucial role in the regulation of Kir6 gating and trafficking (Chan et al. 2003; Fang et al. 2006). TMD0 is also involved in a tight physical interaction with the Kir6 subunit. Besides TMD0, another portion of SUR – a region linking helix 17 with the NBD2 domain –has been reported to interact physically with the pore subunit (Rainbow et al. 2004).

While two isoforms of the human Kir6 protein exists (Kir6.1 and 6.2), three isoforms of the SUR protein are known. SUR1 is mostly expressed in pancreatic *β*-cells and neurons mainly with Kir6.2, SUR2A is abundant in cardiac and skeletal muscles mainly with Kir6.2, and SUR2B is found in smooth muscle mostly with Kir6.1 (Inagaki et al. 1995b). Although the SUR isoforms share a very high level of homology, they confer specific properties to K-ATP complexes. As an example, pancreatic SUR1-Kir6.2 channels are partly active at rest while cardiac SUR2A-Kir6.2 channels are mostly closed, a divergence that could be related to differences in the way SUR isoforms are coupled with Kir6.2.

Despite a considerable amount of literature on the SUR-Kir6.2 physical interaction, very little is known on the mechanisms underlying the functional coupling between these subunits, that is, how binding of drugs or MgADP onto SUR is transduced into gating of Kir6.2. Interestingly, alignment of SUR sequences with other ABC family members reveals a high sequence similarity with the multidrug resistance-associated proteins (MRP) which do not physically and functionally interact with Kir6.2. Taking advantage of this similarity, we previously designed a chimeric strategy to study the role of specific regions of SUR in K-ATP channel function and discovered that three residues within the region linking helix 17 and the NBD2 domain are involved in the functional coupling between SUR2A and Kir6.2 (Dupuis et al. 2008). In order to determine whether a similar mechanism is conserved in other isoforms, we extended here our investigations to SUR1-based channels. Our results show that the same residues previously identified in SUR2A are also involved in the functional coupling between SUR1 and Kir6.2. However, dissimilarities were observed in the properties of their side chains suggesting discreet differences in the activation of Kir6.2 by both SUR1 and SUR2A.

## Methods

### Ethical approval

Animal handling and experiments fully conformed with French regulations and were approved by governmental veterinary services (authorization No. 28-03-15 from the Ministère de l'Agriculture, Direction des Services Vétérinaires to Michel Vivaudou).

### Molecular biology

Experimental conditions were essentially as previously described (Moreau et al. 2005a; Dupuis et al. 2008). All constructs were derived from mouse Kir6.2 (GenBank accession No. D50581), hamster SUR1 (GenBank accession No. Q09427) and human MRP1 (GenBank accession No. L05628) and subcloned in *Xenopus* oocyte expression vectors derived from pGEMHE (Liman et al. 1992). Mutations were introduced by PCR using the QuickChange Site-Directed Mutagenesis Kit (Stratagene, Marcy L'Etoile, France) and the coding sequences of each construct were entirely verified by sequencing. The exact amino acid composition of SUR1-MRP1 chimeric constructs and mutants were as follows: SUR1S1M = SUR1(M1-V1313) + MRP1(V1261-F1341) + SUR1(R1394-K1582); SUR1S2M = SUR1(M1-P1336) + MRP1(P1284-L1300) + SUR1(V1352-K1582); SUR1S3M = SUR1 with mutations N1338S, I1345V, S1351C, and V1352L; SUR1S4M = SUR1 with mutations K1337S, D1341Q, K1344R, Q1346E, and Q1348R; SUR1_(QIL/VFY)_ = SUR1 with mutations Q1342V, I1347F, and L1350Y, SUR1S2M_(VFY/QIL)_ = SUR1S2M with mutations V1290Q, F1295I, and Y1298L, SUR1_(QIL/III)_ = Q1342I, I1347I, and L1350I, SUR1_(QIL/AAA)_ = Q1342A, I1347A, and L1350A, SUR1_(QIL/GGG)_ = Q1342G, I1347G, and L1350G, SUR2A_(EIL/III)_ = E1305I, I1310I, and L1313I, SUR2A_(EIL/AAA)_ = E1305A, I1310A, and L1313A, SUR2A_(EIL/GGG)_ = E1305G, I1310G, and L1313G.

After amplification and linearization, plasmid DNAs were transcribed in vitro using the T7 mMessage mMachine Kit (Life Technologies, Saint Aubin, France) to produce cRNAs for later *Xenopus* oocyte microinjection.

### Oocyte preparation and microinjection

Oocytes were surgically harvested from female *Xenopus laevis*. One animal was operated on each week.

Female *X. laevis* were anesthetized with 3-aminobenzoic acid ethyl ester (1 g/L) for ˜20 min. A minilaparotomy was then performed. *Xenopus laevis* oocytes were defolliculated by 120-min incubation at 19°C in a 2 mg mL^−1^ type A Collagenase solution (Sigma-Aldrich, Saint Quentin Fallavier, France). Stage V or VI selected oocytes were injected the next day with mRNAs encoding wild-type Kir6.2 (2 ng) and wild type or chimeric SURs (6 ng). Injected oocytes were then stored at 19°C in Barth's solution (KCl 1 mmol/L, MgSO_4_ 0.82 mmol/L, NaCl 88 mmol/L, NaHCO_3_ 2.4 mmol/L, CaCl_2_ 0.41 mmol/L, Ca(NO_3_)_2_ 0.3 mmol/L, HEPES (4-(2-Hydroxyethyl)piperazine-1-ethanesulfonic acid) 16 mmol/L, pH 7.4) supplemented with 100 U mL^−1^ penicillin and 100 *μ*g mL^−1^ streptomycin.

### Electrophysiology

Three to 5 days after injection, oocytes were manually devitellinized and channels were characterized by the patch-clamp technique in the excised inside-out configuration at room temperature using a RK300 amplifier (Bio-logic, Claix, France) and a Digidata 1322A acquisition system with Axoscope 9 software (Axon Instruments, Sunnyvale, CA). Patch pipettes contained: 154 mmol/L K^+^, 146 mmol/L Cl^−^, 5 mmol/L Mg^2+^, and 10 mmol/L PIPES-KOH (1,4-Piperazinediethanesulfonic acid - KOH) (pH 7.1). The cytoplasmic face of the patch was bathed in solutions containing 154 mmol/L K^+^, 40 mmol/L Cl^−^, 1 mmol/L EGTA (ethylene glycol-bis(2-aminoethylether)-N,N,N′,N′-tetraacetic acid), 1 mmol/L Mg^2+^, 10 mmol/L PIPES-KOH (pH 7.1), and methanesulfonate as the remaining anion. ATP, ADP, Diazoxide, and P1075 were added as specified. Membrane potential was held at −50 mV during all experiments. Application of various solutions to the patch was performed using a RSC-100 automated perfusion pipes system (Bio-Logic; Vivaudou and Forestier 1995). Pipe switching time was set at 250 msec. Data acquisition and analysis were performed using in-house software. Baseline fluctuations were removed by interactive fitting with a spline curve and subtraction of this fit with the signal. Nonlinear curve fitting was performed with in-house software. The following standard Hill equations were used for fitting.

For activation by openers:

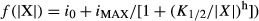
1where 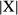 is the concentration of activator, *i*_0_ is the control normalized current in the absence of activator, *i*_Max_ is the maximal activator-induced current, *K*_½_ the concentration for half-maximal activation, and h the Hill coefficient.

For inhibition by nucleotides:

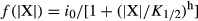
2where 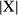 is the concentration of inhibitor, *i*_0_ is the control normalized current in the absence of inhibitor, *K*_½_ the concentration for half-maximal inhibition, and h the Hill coefficient.

Results are displayed as mean ± SEM. Error bars in figures represent SEM and are only shown if greater than symbols.

## Results

### Residues Q1342, I1347, and L1350 of SUR1 are essential in transducing activation by Diazoxide and ADP to Kir6.2

We previously identified three residues in the region linking the TMD2 and NBD2 domains of SUR2A as important determinants of the coupling between SUR2A and Kir6.2. Mutating these residues prevented KCO- and ADP-induced activation of the channel without affecting the affinity of SUR2A for both activator families. In this study, a similar chimeric strategy was applied to the SUR1 isoform in order to identify the key residues involved in the activation of Kir6.2.

**Figure 2:**
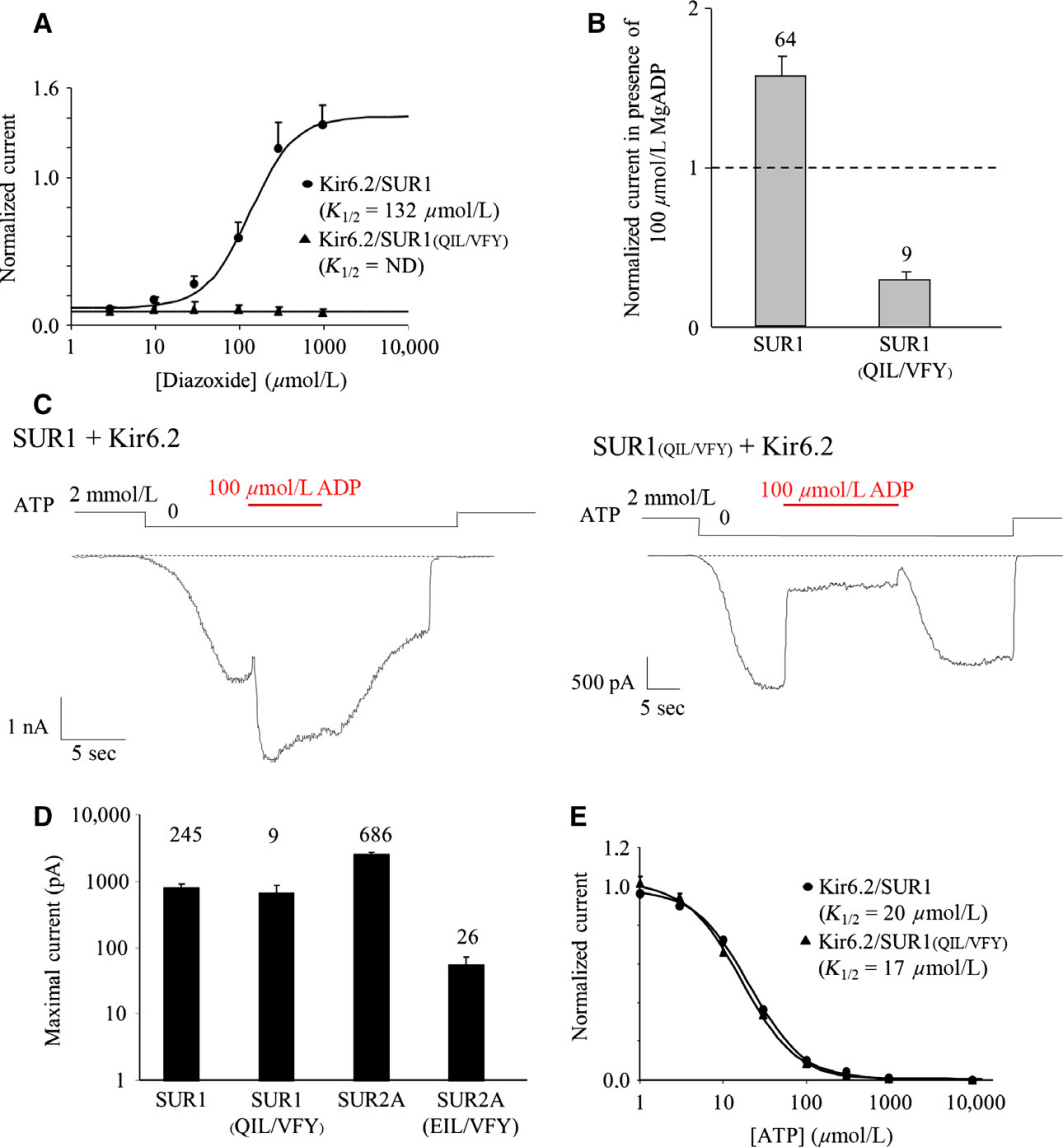
Characterization of the SUR1_(__QIL__/__VFY__)_ mutant. (A) Diazoxide dose–response relationships for Kir6.2/SUR1 (circles) and Kir6.2/SUR1_(__QIL__/__VFY__)_ (triangles). Currents were normalized to the current measured in 0 ATP. Hill equation fitting yielded *K*_1/2_ = 132 *μ*mol/L (h = 1.65) for Kir6.2/SUR1. (B) Response to 100 *μ*mol/L MgADP of SUR1 wt and SUR1_(__QIL__/__VFY__)_. Currents were normalized to the current measured in the absence of nucleotides immediately before MgADP application. Numbers indicate the number of patches included in each average. (C) Representative patch-clamp recordings illustrating the responses of wild type and SUR1(QIL/VFY) channels to 100 *μ*mol/L MgADP in the absence of ATP. (D) Current amplitudes in the absence of nucleotides of Kir6.2/SUR1, Kir6.2/SUR1_(__QIL__/__VFY__)_, Kir6.2/SUR2A, and Kir6.2/SUR2A_(__EIL__/__VFY__)_. (E) ATP dose–response relationships for Kir6.2/SUR1 (circles) and Kir6.2/SUR1_(__QIL__/__VFY__)_ (triangles). Currents were normalized to the current measured in 0 ATP. Hill equation fitting yielded *K*_1/2_ = 20 *μ*mol/L (h = 1.33) for Kir6.2/SUR1 and *K*_1/2_ = 17 *μ*mol/L (h = 1.32) for Kir6.2/SUR1_(__QIL__/__VFY__)_. SUR1, sulfonylurea receptor 1.

Using inside-out patch clamp recordings, MRP1/SUR1 chimeras coexpressed with Kir6.2 in *Xenopus* oocytes were first tested for their response to openers (Diazoxide, 300 *μ*mol/L and MgADP, 100 *μ*mol/L). As described in Figure 1, we were able to measure Kir6.2-generated currents from all constructs except MRP1 coexpressed with Kir6.2, as expected and previously reported (Dupuis et al. 2008). The SUR1S1M chimera, in which SUR1 residues 1313–1394 were replaced by the corresponding amino acids of MRP1, showed no response to Diazoxide (Fig. 1D) although it was still able to traffick to the plasma membrane, suggesting the presence of critical SUR1 residues in this region for Diazoxide-evoked activation of Kir6.2. In order to identify those residues, we progressively replaced shorter regions of SUR1 by the corresponding residues of MRP1. Chimera SUR1S2M, in which central residues of the region P1336-V1352 were exchanged, displayed also an altered response to Diazoxide, locating the critical residues in this 16 residue-long region. Further refinements were performed by point mutations as shown in Figure 1B and C for the chimeras SUR1S3M (4 residues), SUR1S4M (5 residues) and SUR1_(QIL/VFY)_. For SUR1S3M and SUR1S4M, Diazoxide application produced a robust activation equivalent to that observed with wild-type SUR1. Consequently, the mutated residues are not essential for the Diazoxide-evoked regulation. In contrast, replacement of the SUR1 QIL residues by the MRP1 VFY residues (SUR1_(QIL/VFY)_, Fig. 1D and E) abolished the activation by the opener indicating that those residues are essential for the Diazoxide-induced activation of Kir6.2. To verify that the QIL residues are also sufficient to restore the Diazoxide action, the reverse MRP1 chimera was created by reintroducing the SUR1 QIL residues in the MRP1 sequence of the SUR1S2M chimera (SUR1S2M_(VFY/QIL)_). Application of Diazoxide induced an activation confirming that the SUR1 QIL residues are not only essential but also sufficient for activation by Diazoxide. As shown in Figure 2A, Diazoxide had small effects on SUR1_(QIL/VFY)_, even at a saturating concentration of 1 mmol/L. Involvement of those residues in the activation of Kir6.2 by the physiological opener MgADP was also assessed and the results, shown in Figure 2B and C, were equivalent to the results obtained with Diazoxide. Noteworthy, the SUR1_(QIL/VFY)_/Kir6.2 channels were strongly inhibited by MgADP (70% inhibition) while the wild type is activated (53% activation). Such observation is in line with the antagonistic effects of ADP, which causes inhibition by binding to Kir6.2 and activation by binding to SUR (Hosy and Vivaudou 2014). These results suggest that, in SUR1, residues Q1342, I1347, and L1350 are required to transmit activation from SUR1 to Kir6.2. These residues correspond to those previously reported (EIL) for the SUR2A subunit (Fig. 1B) (Dupuis et al. 2008). However, a difference was observed between SUR2A and SUR1 residues as the basal current of SUR1_(QIL/VFY)_ was 12-fold higher than SUR2A_(EIL/VFY)_ (Fig. 2D) suggesting that the MRP1 VFY residues disturbed less the SUR1/Kir6.2 interaction than the SUR2A/Kir6.2 and yielded a stronger surface expression. The higher current amplitude for SUR1_(QIL/VFY)_ was not due to a lesser ATP sensitivity of the channel because dose-dependent inhibition by ATP was unaffected by the mutations, with IC_50_ = 17 *μ*mol/L versus 20 *μ*mol/L for wild-type channels (Fig. 2E).

### Differences in the coupling of Kir6.2 with SUR1 and SUR2A revealed by alanine mutants

**Figure 3:**
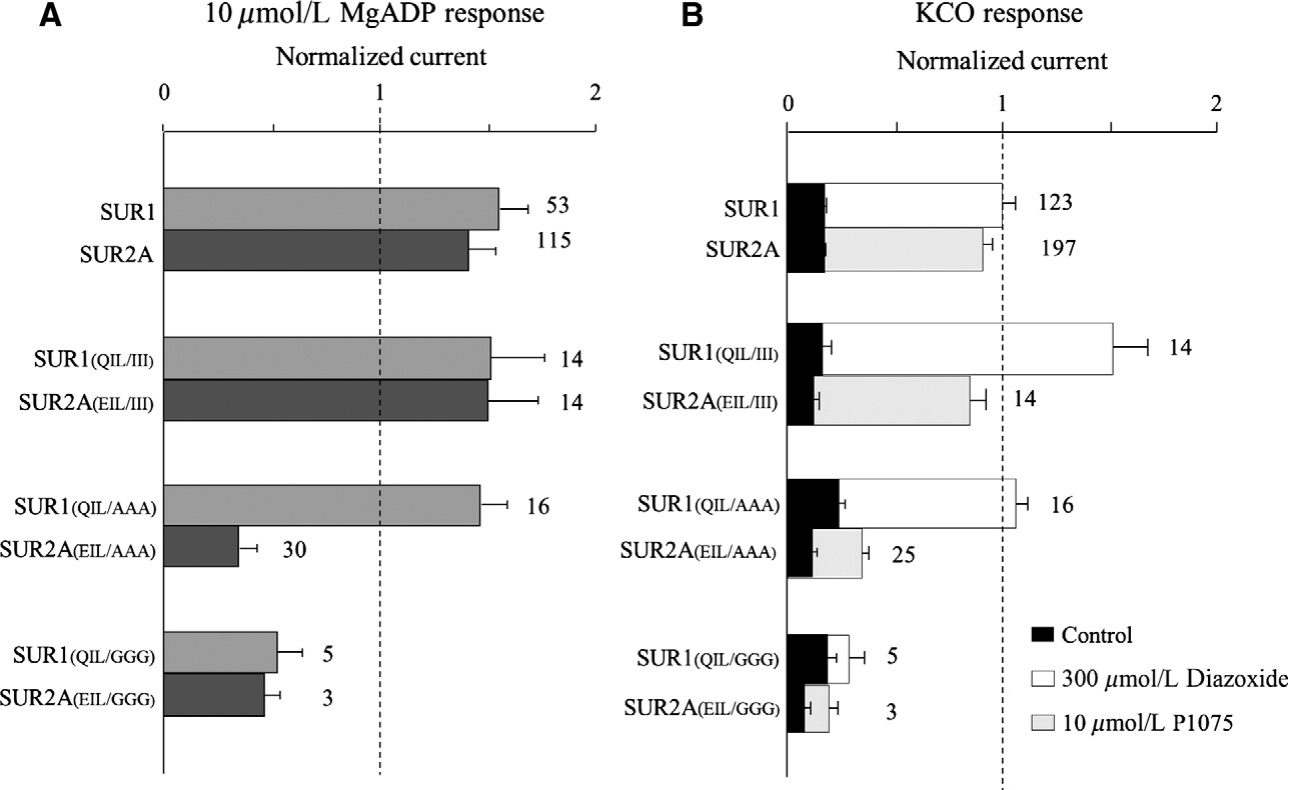
Openers response by sulfonylurea receptor 1 (SUR1) and SUR2A wt and relative mutants. Residues Q1342, I1347, and L1350 of SUR1, and the matching residues E1305, I1310, and L1313 of SUR2A were mutated into alanine, glycine, and isoleucine. (A) The effects of the application of 100 *μ*mol/L MgADP were measured in inside-out patches excised from oocytes coexpressing Kir6.2 and the indicated SUR constructs. Currents were normalized to the current measured in the absence of nucleotides immediately before MgADP application. Numbers at right of bars indicate the number of patches included in each average. (B) The effects of 300 *μ*mol/L Diazoxide or 10 *μ*mol/L P1075 were measured in inside-out patches excised from oocytes. Diazoxide (300 *μ*mol/L) was applied in the presence of 100 *μ*mol/L ATP. P1075 (10 *μ*mol/L) was applied in the presence of 100 *μ*mol/L ATP. Control designates the current measured in 100 *μ*mol/L ATP before application of the tested opener.

The SUR1 QIL and SUR2A EIL residues appear necessary and sufficient for the activation of Kir6.2 by SUR ligands. To further investigate the role of these residues, we explored their molecular specificity by mutating them to the following amino acids: Ile, Gln, Glu, Ala, Gly. Ile is hydrophobic with a long side chain; Gln is an uncharged polar residue at pH 7 able to create hydrogen bonds; Glu is a negatively charged amino acid, while Ala is hydrophobic with a short side chain and Gly has no side chain. All mutants were tested for their response to MgADP and pharmacological openers. Mutations of the critical residues to Gln or Glu resulted in the loss of MgADP activation for both SUR isoforms, indicating that the hydrophobicity of these residues I and L is essential for MgADP action (Fig. S1). The critical role of these residues is emphasized by the mutants SUR1_(QIL/III)_ and SUR2A_(EIL/III)_ which are still activated by MgADP and the pharmacological openers (Fig. 3). Mutations to Gly (SUR1_(QIL/GGG)_ and SUR2A_(EIL/GGG)_) abolished the activation by openers, suggesting that the presence of the lateral chains is required. Unexpectedly, mutations to Ala (SUR1_(QIL/AAA)_ and SUR2A_(EIL/AAA)_) yielded distinct responses to openers, SUR1_(QIL/AAA)_ is still activated whereas SUR2A_(EIL/AAA)_ was not. Altogether, these results suggest that similar residues in SUR1 and SUR2A seem to be involved in the regulation of Kir6.2 by SUR, but they also contribute to isoform-specificity of the coupling mechanisms.

## Discussion

**Figure 4:**
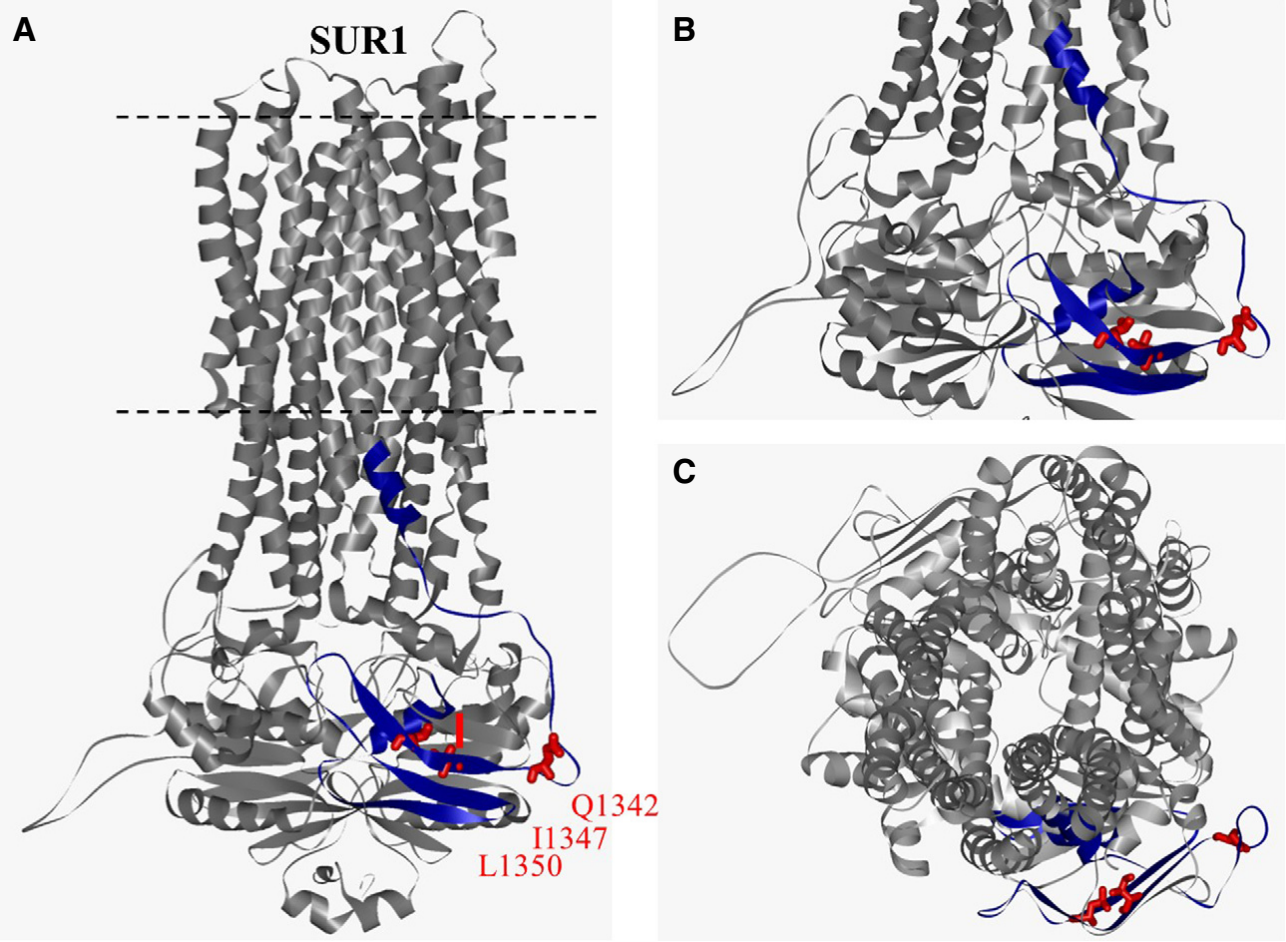
Sulfonylurea receptor 1 (SUR1) homology model based on multidrug resistance protein 1 (Bessadok et al. 2011). (A) Lateral view. The region identified by Rainbow et al. (2004) is highlighted in dark blue (SUR1S1M fragment). Residues Q1342, I1347, and L1350 are represented in ball-and-stick format and colored in red. (B) Close-up lateral view of the cytoplasmic domain. (C) axial view from the extracellular side.

Using a structure-function approach, we identified crucial residues in the SUR1 subunit responsible for allosteric upregulation of Kir6.2. Indeed, mutations of SUR1 residues Q1342, I1347, and L1350 into the corresponding residues (VFY) of MRP1 prevented K-ATP channel activation by both MgADP and Diazoxide. Mutagenetic analysis of these three residues revealed the critical role of the hydrophobic residues for the activation of the channel by openers. Surprisingly, Alanine mutations in the context of SUR1 and SUR2A had opposite impact on the SUR-mediated regulation of Kir6.2, suggesting subtle differences in the molecular mechanisms occurring in the two isoforms (Fig. 3). Because the three identified residues are predicted to be part of the NBD2 domain (Fig. 4), their mutation could affect the binding of the openers, leading to an absence of activation by MgADP and KCOs. While this hypothesis cannot be excluded, it is not supported by the unchanged ATP sensitivity of the mutants. Indeed, ATP dose–response experiments performed in the absence of MgADP demonstrated that nucleotide inhibition of Kir6.2 was similar in the presence of wild-type SUR1 and in that of the mutant SUR1_(QIL-VFY)_ (Fig. 2E). Because the binding site for Diazoxide has not been precisely identified, we cannot exclude the hypothesis that it is affected by the triple mutations. However, it is notorious that Diazoxide-activation is dependent on MgADP binding to NBD2 (Gribble et al. 1997; Schwanstecher et al. 1998; D'hahan et al. 1999). Moreover, since the responses to Diazoxide and ADP are similarly impaired in both isoforms (Dupuis et al. 2008) the likely interpretation of our results is that mutating Q1342V, I1347F, and L1350Y affects the communication pathway from SUR1 to Kir6.2 rather than the binding of the effectors to SUR1.

Moreover, residue L1350 has been previously correlated with a mutation (L1350Q) that causes congenital hyperinsulinism by preventing surface expression of K-ATP channels in pancreatic *β*-cells (Yan et al. 2007). While we tested the functionality of this mutant, we did not observe any difference with the wild type when comparing its activation ability by ADP (Fig. S1) and Diazoxide or its ATP sensitivity (data not shown). Interestingly, comparison of the mutant SUR1_(QIL/QQQ)_ with the mutant SUR1_(L1350Q)_ which is equivalent to SUR1_(QIL/QIQ)_ reveals that mutation of a single residue is not sufficient to disturb the activation as observed with SUR2A (Dupuis et al. 2008). All single mutants displayed wild-type channel properties while most of the double mutations led to an intermediate phenotype, which suggests that the combination of these three residues rather than each individual residue itself is crucial to signal transduction between SUR1 and Kir6.2 subunits. Mutations in other residues such as Glu and Gly also affect the MgADP activation, but unexpectedly, mutations to Ala preserve the wild-type phenotype. Altogether these results indicate the critical role of these residues which can be replaced by a residue with short and hydrophobic side chain (Ala) but not by hydrophilic (Q and E), nor side-chain-free residue (Gly), nor large hydrophobic residue (Phe). Similar mutations in SUR2A residues caused equivalent phenotype, except for mutations to Alanine that abolished the activation by MgADP. This unexpected SUR isoform difference suggests that the nature of the side chain is more critical for SUR2A activity than for SUR1. This observation could be explained by at least two hypotheses. In a first scenario, the interaction between SUR subunits and Kir6.2 could occur in different position depending on the global flexibility of the studied region proximal to NBD2. A second hypothesis would be a difference in the NBD2 activity of SUR1 and SUR2A which has been observed in structure-function studies of the C-terminal domain of SUR isoforms (de Wet et al. 2010). It is still not understood why, in physiological conditions in the resting state, pancreatic SUR1 + Kir6.2 channels are partially opened while cardiac SUR2A + Kir6.2 channels are mostly closed whereas they share similar ATP and ADP sensitivities in excised patch recordings. Could these residues be implicated in this difference? Maybe a difference in the activation mechanism of SUR isoforms could be implicated in this essential physiological difference.

In the absence of high-resolution structure of the K-ATP channel, we used a model of SUR1 published by Bessadok et al. (2011) to spatially locate the residues QIL of SUR1 (Fig. 4). Residue Q1342 is predicted to stand in a loop just before a *β*-sheet of the NBD2 which includes I1347 and L1350. Interestingly, a novel interaction between the NBD2 proximal C-terminus of SUR2A and the cytoplasmic C-terminal domain of Kir6.2 was recently identified (Lodwick et al. 2014). According to the model, SUR2A-Glu1318 is located within the NBD2 domain, just few residues away from those identified by Dupuis et al. (2008). SUR2A-Glu1318 and Kir6.2-Lys338 form a salt bridge responsible for the transmission of allosteric changes to Kir6.2. To note, this model also highlights another amino acid, F1388, which can interact and form a tightly packed hydrophobic core with residues I1347 and L1350, connecting the *β*-sheet interface including I1347 and L1350 with the *α*-helical region of NBD2 which comprises part of its Walker A motif. Interestingly, deletion of F1388 alters the trafficking but also the function of K-ATP channels and is associated with PHHI (Cartier et al. 2001). Thus, changing the nature of the side chain of I1347 and L1350 could have an impact on the channel sensitivity toward ADP and KCOs. This is emphasized by the observation that mutation of another hydrophobic residue (I) within QIL altered less the channel activity than that of polar residues (Q and E). According to this model, residues Q1342, I1347, and L1350 of SUR1 (and the corresponding residues in SUR2A) could stand at the interface between SURs and Kir6.2 and thereby be part of a mechanism of allosteric regulation of the pore-forming subunit.

## Conflict of Interest

None declared.

## Supporting Information

**Figure S1.** Relative responses to 100 *μ*mol/L MgADP of SUR1 and SUR2A wt and mutants. Currents were normalized to the current measured in the absence of nucleotides immediately before MgADP application. Numbers indicate the number of patches included in each average.

## References

[b1] Aguilar-Bryan L, Nichols CG, Wechsler SW, Clement JP, Boyd AE, González G (1995). Cloning of the beta cell high-affinity sulfonylurea receptor: a regulator of insulin secretion. Science.

[b2] Ashcroft FM, Ashcroft SJ, Harrison DE (1988). Properties of single potassium channels modulated by glucose in rat pancreatic beta cells. J. Physiol.

[b3] Bessadok A, Garcia E, Jacquet H, Martin S, Garrigues A, Loiseau N (2011). Recognition of sulfonylurea receptor (ABCC8/9) ligands by the multidrug resistance transporter P-glycoprotein (ABCB1): functional similarities based on common structural features between two multispecific ABC proteins. J. Biol. Chem.

[b4] Cartier EA, Conti LR, Vandenberg CA, Shyng SL (2001). Defective trafficking and function of KATP channels caused by a sulfonylurea receptor 1 mutation associated with persistent hyperinsulinemic hypoglycemia of infancy. Proc. Natl. Acad. Sci. USA.

[b5] Chan KW, Zhang H, Logothetis DE (2003). N-terminal transmembrane domain of the SUR controls trafficking and gating of Kir6 channel subunits. EMBO J.

[b6] Clement JP, Kunjilwar K, Gonzalez G, Schwanstecher M, Panten U, Aguilar-Bryan L (1997). Association and stoichiometry of KATP channel subunits. Neuron.

[b7] D'hahan N, Moreau C, Prost AL, Jacquet H, Alekseev AE, Terzic A (1999). Pharmacological plasticity of cardiac ATP-sensitive potassium channels toward diazoxide revealed by ADP. Proc. Natl. Acad. Sci. USA.

[b8] Dupuis JP, Revilloud J, Moreau CJ, Vivaudou M (2008). Three C-terminal residues from the sulphonylurea receptor contribute to the functional coupling between the KATP channel subunits SUR2A and Kir6.2. J. Physiol.

[b9] Fang K, Csanády L, Chan KW (2006). The N-terminal transmembrane domain (TMD0) and a cytosolic linker (L0) of sulphonylurea receptor define the unique intrinsic gating of KATP channels. J. Physiol.

[b10] Gribble FM, Ashcroft FM (1999). Differential sensitivity of beta-cell and extrapancreatic KATP channels to gliclazide. Diabetologia.

[b11] Gribble FM, Tucker SJ, Ashcroft FM (1997). The essential role of the Walker A motifs of SUR1 in KATP channel activation by Mg-ADP and diazoxide. EMBO J.

[b12] Hosy E, Vivaudou M (2014). The unusual stoichiometry of ADP activation of the KATP channel. Front. Physiol.

[b13] Inagaki N, Tsuura Y, Namba N, Masuda K, Gonoi T, Horie M (1995a). Cloning and functional characterization of a novel ATP-sensitive potassium channel ubiquitously expressed in rat tissues, including pancreatic islets, pituitary, skeletal muscle, and heart. J. Biol. Chem.

[b14] Inagaki N, Gonoi T, Clement JP, Namba N, Inazawa J, Gonzalez G (1995b). Reconstitution of IKATP: an inward rectifier subunit plus the sulfonylurea receptor. Science.

[b15] Kane GC, Liu XK, Yamada S, Olson TM, Terzic A (2005). Cardiac KATP channels in health and disease. J. Mol. Cell. Cardiol.

[b16] Liman ER, Tytgat J, Hess P (1992). Subunit stoichiometry of a mammalian K+ channel determined by construction of multimeric cDNAs. Neuron.

[b17] Lodwick D, Rainbow RD, Rubaiy HN, Al Johi M, Vuister GW, Norman RI (2014). Sulphonylurea receptors regulate the channel pore in ATP-sensitive potassium channels via an inter-subunit salt bridge. Biochem. J.

[b18] Mikhailov MV, de Campbell JD, Wet H, Shimomura K, Zadek B, Collins RF (2005). 3-D structural and functional characterization of the purified KATP channel complex Kir6.2-SUR1. EMBO J.

[b19] Moreau C, Jacquet H, Prost AL, D'hahan N, Vivaudou M (2000). The molecular basis of the specificity of action of KATP channel openers. EMBO J.

[b20] Moreau C, Gally F, Jacquet-Bouix H, Vivaudou M (2005a). The size of a single residue of the sulfonylurea receptor dictates the effectiveness of K ATP channel openers. Mol. Pharmacol.

[b21] Moreau C, Prost AL, Dérand R, Vivaudou M (2005b). SUR, ABC proteins targeted by KATP channel openers. J. Mol. Cell. Cardiol.

[b22] Nichols CG, Shyng SL, Nestorowicz A, Glaser B, Clement JP, Gonzalez G (1996). Adenosine diphosphate as an intracellular regulator of insulin secretion. Science.

[b23] Noma A (1983). ATP-regulated K+ channels in cardiac muscle. Nature.

[b24] Rainbow RD, James M, Hudman D, Al Johi M, Singh H, Watson PJ (2004). Proximal C-terminal domain of sulphonylurea receptor 2A interacts with pore-forming Kir6 subunits in KATP channels. Biochem. J.

[b25] Schwanstecher M, Sieverding C, Dörschner H, Gross I, Aguilar-Bryan L, Schwanstecher C (1998). Potassium channel openers require ATP to bind to and act through sulfonylurea receptors. EMBO J.

[b26] Seino S, Miki T (2003). Physiological and pathophysiological roles of ATP-sensitive K+ channels. Prog. Biophys. Mol. Biol.

[b27] Vivaudou M, Forestier C (1995). Modification by protons of frog skeletal muscle KATP channels: effects on ion conduction and nucleotide inhibition. J. Physiol.

[b28] de Wet H, Fotinou C, Amad N, Dreger M, Ashcroft FM (2010). The ATPase activities of sulfonylurea receptor 2A and sulfonylurea receptor 2B are influenced by the C-terminal 42 amino acids. FEBS J.

[b29] Yan FF, Lin YW, MacMullen C, Ganguly A, Stanley CA, Shyng SL (2007). Congenital hyperinsulinism associated ABCC8 mutations that cause defective trafficking of ATP-sensitive K+ channels: identification and rescue. Diabetes.

[b30] Zerangue N, Schwappach B, Jan YN, Jan LY (1999). A new ER trafficking signal regulates the subunit stoichiometry of plasma membrane KATP channels. Neuron.

