# Supplementary figures and images for: Kir6.2 activation by sulfonylurea receptors: a different mechanism of action for SUR1 and SUR2A subunits via the same residues
Source: Physiol Rep. 2015 Sep 28;3(9):e12533. doi: 10.14814/phy2.12533 (PMC4600379; doi:10.14814/phy2.12533)

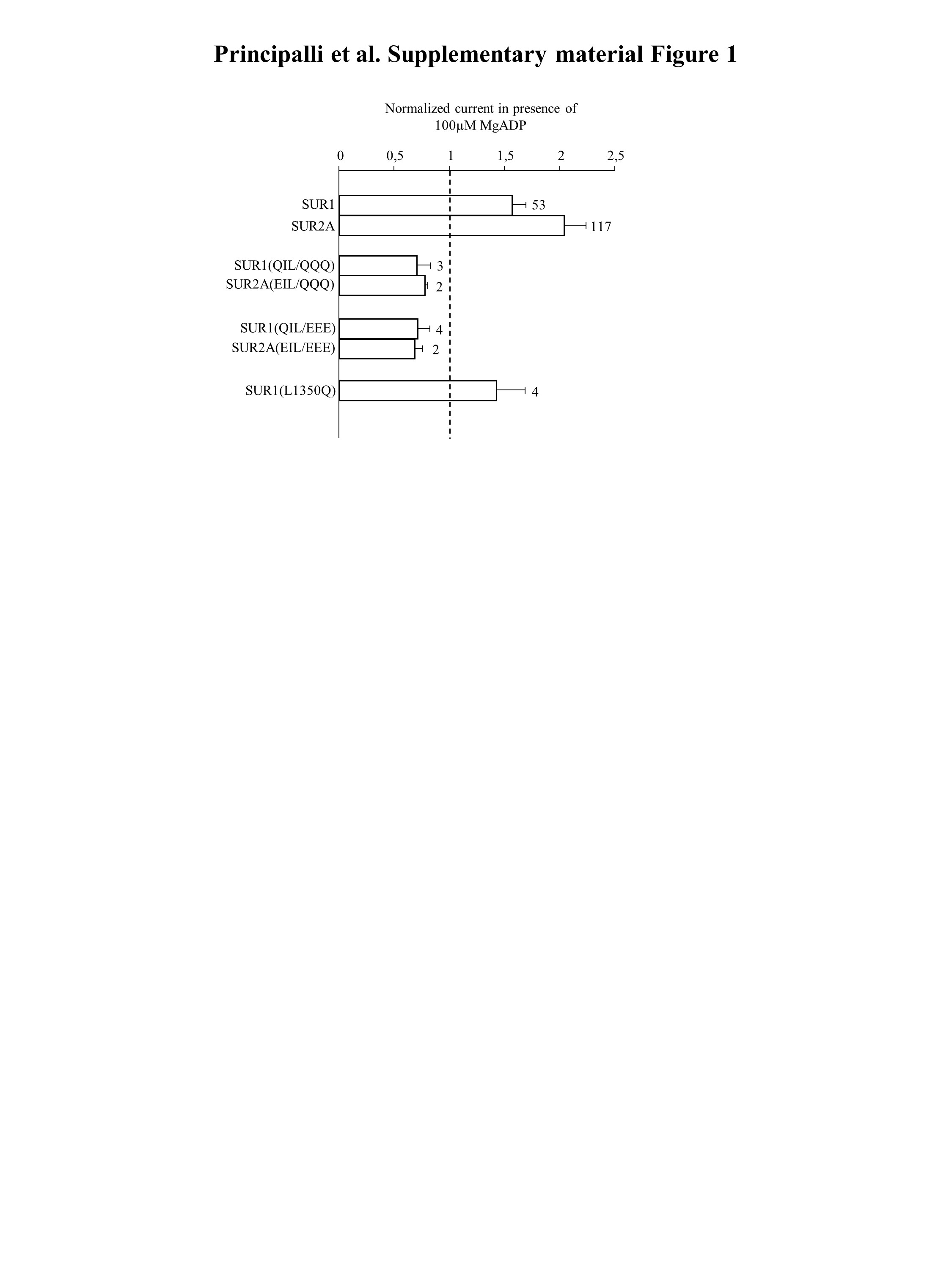

Supplement: Supplementary file 1 [file phy20003-e12533-sd1.gif]
